# Performance Optimization for a Class of Petri Nets

**DOI:** 10.3390/s23031447

**Published:** 2023-01-28

**Authors:** Weijie Shi, Zhou He, Chan Gu, Ning Ran, Ziyue Ma

**Affiliations:** 1 School of Electro-Mechanical Engineering, Shaanxi University of Science and Technology, Xi’an 710021, China; 2 School of Electrical and Control Engineering, Shaanxi University of Science and Technology, Xi’an 710021, China; 3 College of Electronic and Information Engineering, Heibei University, Baoding 071002, China; 4 School of Electro-Mechanical Engineering, Xidian University, Xi’an 710071, China

**Keywords:** flexible manufacturing systems, Petri nets, evolutionary techniques, resource allocation

## Abstract

Petri nets (PNs) are widely used to model flexible manufacturing systems (FMSs). This paper deals with the performance optimization of FMSs modeled by Petri nets that aim to maximize the system’s performance under a given budget by optimizing both quantities and types of resources, such as sensors and devices. Such an optimization problem is challenging since it is nonlinear; hence, a globally optimal solution is hard to achieve. Here, we developed a genetic algorithm combined with mixed-integer linear programming (MILP) to solve the problem. In this approach, a set of candidate resource allocation strategies, i.e., the choices of the number of resources, are first generated by using MILP. Then, the choices of the type and the cycle time of the resources are evaluated by MILP; the promising ones are used to spawn the next generation of candidate strategies. The effectiveness and efficiency of the developed methodology are illustrated by simulation studies.

## 1. Introduction

Performance optimization in flexible manufacturing systems has received great attention in recent decades [1]. Such automated systems with synchronization, concurrency, and time delay can usually be modeled by timed marked graphs (TMGs), an important subclass of PNs that are proven to be useful in modeling manufacturing systems, queue systems, and railway transportation systems [2,3,4,5,6,7,8,9,10]. The performance of a system modeled with TMGs is usually characterized by the cycle time that represents the normalized time for one cycle of production. This issue was first studied in ordinary TMG (i.e., TMGs in which the weights on the arcs are unitary). In the literature, ordinary TMGs are simply called “TMGs” by omitting the term “ordinary”. The cycle time can be obtained by exploring the periodic dynamic evolution of a TMG [11]. On the other hand, the exact cycle time of a TMG can also be computed analytically by using the linear programming technique [12]. Algebraic approaches based on (max,+) or (min,+) algebra have also been applied for computing the cycle time of TMGs [13]. Based on these results, various methods have been developed for the performance optimization problems in TMGs, i.e., to minimize the total cost of the system’s resources (machines, devices, tasks, etc.) without compromising the desired level of throughput. The optimization targets include the initial resource allocation [14,15,16,17,18,19,20] and the server-type selection under a given budget [21,22,23].

Timed weighted marked graphs (TWMGs) are more general than ordinary TMGs and are useful in modeling systems with batch processes (i.e., instead of processing tasks one by one, they wait until a batch of tasks are available and process them simultaneously) [24,25]. Nevertheless, obtaining the exact cycle time of a TWMG is much more complicated than that of a TMG. In [26], the authors prove that for a given TWMG, the optimization of the server type is NP-hard. A widely used method for computing the cycle time of a TWMG is to first transform it into an equivalent TMG [27,28] followed by the linear programming technique in [12]. However, such a transformation is computationally heavy, initially marking-dependent, and usually yields a TMG much larger than the original TWMG. To overcome such a problem, in [29], a method is proposed to transform a TWMG (with an undetermined initial marking) into an equivalent parametric TMG so that the scale of the corresponding linear programming remains solvable. Some approximation methods [30] are also developed to estimate the upper bound of the cycle time of a TWMG. A linearization method for TWMGs to obtain linear representation in (min,+) was developed [31]. Based on the obtained linear model, the performance of the TWMGs can be analyzed.

The performance optimization problem in TWMGs is also more complex than that of TMGs. Relatively few works have been conducted on the performance optimization of TWMGs in the literature. Since the equivalent TMG classes of a TWMG is finite, by solving a mixed-integer linear programming problem (MILPP) for each equivalent TMG class, a globally optimal initial marking can be obtained [32,33]. Nevertheless, the computational load of this precise algorithm is unsatisfactory, since the number of equivalent TMG classes is exponential in the number of places in the corresponding TWMG. Therefore, people turn to hybrid algorithms, such as heuristics combined with simulation and MILPPs, which are fast and provide near-optimal solutions [34,35,36]. In [34], a TWMG is initialized with a sufficiently large initial marking so that the performance requirement is satisfied. Then, a greedy algorithm is applied to decrease the total cost in which tokens are iteratively removed until the cycle time reaches a lower bound threshold. A heuristic method combined with integer linear programming was proposed in [35]. However, both heuristics in [34,35] rely on the detection of the so-called critical places and, hence, a time-consuming simulation of the TWMG has to be performed in each iteration. This method is further improved in [36] in which the critical places are determined by linear programming, which greatly improves the efficiency of the heuristic algorithm. Computational efficient solutions for the performance optimization of TWMGs are still missing.

In practice, the execution time of a process may vary when different types of resources (machine, robot, transporting vehicle, etc.) are deployed. Therefore, the optimization of the types and quantities of resources, (so that the performances are maximized) has also received considerable attention in different areas, such as manufacturing systems [37,38,39] and enterprise systems [40]. However, as far as we know, no work has been done on such a problem in TWMGs. Solving this problem is challenging since it is a nonlinear quadratic optimization (which we will see in Section 3) and, thus, a globally optimal solution is hard to achieve. The search space for selecting the types and quantities of resources is quite huge even though the system size is small.

In this paper, we explore the performance optimization problem in TWMGs where both quantities and types of resources are simultaneously optimized. The exact value of the cycle time of TWMGs is efficiently evaluated by using linear algebraic methods. Precisely speaking, our aim is to minimize the cycle time of a given TWMG subject to a given budget limit, while both quantities and types of resources are variables.

The main contribution of this work is summarized as follows. We developed a hybrid method that is a genetic heuristic combined with MILP. Initially, a set of candidate resource allocation strategies, i.e., the choices of the number of resources, are first generated by solving a MILPP. Then, among all of the possible types of resources whose numbers grow exponentially with respect to the system size, we originally developed a MILPP to determine the optimal one. This was done by transforming the determined TWMG of the candidate strategy into an equivalent TMG with parametric firing delays. In the latter, a MILPP was applied to compute its cycle time and the gross cost (scored as fitness). The promising strategies, i.e., those with high fitness, were used to spawn the next generation of candidate strategies. The procedure above was repeated for a pre-set number of generations, and eventually, a near-optimal resource allocation strategy was obtained. Simulation results show that the developed approach can provide a good trade-off between the near-optimality of the solution and the computational load.

This paper is structured as follows. The basic concepts used in this paper are discussed in Section 2. Section 3 formulates the performance optimization problem studied in this paper. In Section 4, a genetic algorithm-based approach is presented. Simulation studies are investigated in Section 5. Conclusions and future work are presented in Section 6.

## 2. Background

### 2.1. Generalities

A PN is a 4-tuple N=(P,T,Pre,Post), where *P* is a set of *n* places; *T* is a set of *m* transitions; Pre:P×T→N and Post:P×T→N are the pre- and post-incidence functions that specify the arcs, respectively, and are also denoted by matrices in $N^{n\times m}$, where N={0,1,2,…}; $C=\mathrm{Post}-\mathrm{Pre}\in Z^{n\times m}$ is the incidence matrix, where Z={0,±1,±2,…}. A PN is said to be ordinary when all of its arc weights are unitary, i.e., $\mathrm{Pre},\mathrm{Post}\in {\left\{0,1\right\}}^{n\times m}$.

A vector $y={\left(y_{1},y_{2},\ldots ,y_{n}\right)}^{T}\in N^{n}$ (resp., $x={\left(x_{1},x_{2},\ldots ,x_{m}\right)}^{T}\in N^{m}$) such that y≠0 and $y^{T}\cdot C=0$ (resp., x≠0 and C·x=0) is a P-semiflow (resp., T-semiflow). The support of a P-semiflow (resp., T-semiflow) is defined by ∣∣y∣∣=$\left\{p_{i}\in P|y_{i}>0\right\}$ (resp., ∣∣x∣∣=$\left\{t_{i}\in T|x_{i}>0\right\}$). A P-semiflow y (resp., T-semiflow x) is minimal if (i) ∣∣y∣∣ (resp., ∣∣x∣∣) is not a superset of the support of any other P-semiflow (resp., T-semiflow), and (ii) all $y_{i},y_{j}$ (resp., $x_{i},x_{j}$) where i≠j are mutually prime.

The set of output (resp., input ) places of transition $t_{i}\in T$ is defined as $t_{i}^{•}=\left\{p\in P|Post\left(p,t_{i}\right)>0\right\}$ (resp., ${}^{•}t_{i}=\left\{p\in P|Pre\left(p,t_{i}\right)>0\right\}$). The notions for ${}^{•}p$ and $p^{•}$ are analogously defined.

A marking is a mapping M:P→N that assigns to each place of a PN a non-negative integer number of tokens, which is also described as an *n*-component vector $M\in N^{n}$. The number of tokens of place *p* at marking M is denoted by M(p). A Petri net system $\langle N,M_{0}\rangle$ is a net *N* with an initial marking $M_{0}$.

A weighted marked graph (WMG) is a PN in which each place has exactly one input and one output transition. A marked graph (MG) is a WMG whose weights on arcs are unitary.

Given a PN *N*, a path is a sequence of nodes $o_{1}o_{2}\cdots o_{q}$ where $o_{i}\in P\cup T$ for all i∈{1,…,q} and $o_{i+1}\in o_{i}^{•}$ holds for all i∈{1,…,q−1}. A PN is said to be strongly connected if, for any $o,o^{\prime }\in P\cup T$, there exists a path from *o* to $o^{\prime }$. A path $o_{1}o_{2}\cdots o_{q}$ is a circuit if $o_{1}=o_{q}$. A circuit $o_{1}o_{2}\cdots o_{q-1}o_{1}$ is an elementary circuit, denoted by γ, if for all i,j∈{1,…,q}, i≠j indicates $o_{i}\neq o_{j}$. The set of all elementary circuits of *N* is denoted by Γ.

A WMG is neutral if for each elementary circuit γ it holds that $\prod_{p_{j}\in \gamma }\frac{Pre(p_{j},p_{j}^{•})}{Post(p_{j},{}^{•}p_{j})}=1$. In the rest of this paper, we limit our study to neutral and strongly connected WMGs.

### 2.2. Timed Petri Nets

In the literature, two types of timed Petri nets (TPNs) [41] are mainly studied, namely transition-timed PNs (TTPNs) and place-timed PNs (PTPNs) [42]. A transition-timed Petri net is a pair $N_{d}$ = (N,δ) where

- N=(P,T,Pre,Post) is a PN;
- $\delta ={\left[\delta \left(t_{1}\right),\delta \left(t_{2}\right),\ldots ,\delta \left(t_{m}\right)\right]}^{T}\in Q_{\geq 0}^{m}$ is an *m*-component vector, called the firing delay vector of $N_{d}$, which assigns a non-negative rational value to each transition to represent its firing delay, where Q denotes the set of rational numbers.

For a PTPN, a non-negative rational duration δ(p) is assigned to each place *p* to represent the residual time that a token must spend in *p* before it becomes available for its output transitions.

Given a TTPN $N_{d}=\left(N,\delta \right)$, the enabling degree of a transition *t* logically enabled at a marking M, denoted as en(M,t), is the largest integer *r*, such that r·Pre(·,t)≤M. The TTPNs considered in this paper follow the so-called infinite server semantics [42]. In plain words, each transition represents an operation that can be executed as many times as the number of available servers, i.e., each enabled transition can fire as many times as its enabling degree.

A TTPN (N,δ) is called a TWMG if net *N* is a weighted marked graph. A TWMG system is a triple $G=\langle N,\delta ,M_{0}\rangle$, where (N,δ) is a TWMG and $M_{0}$ is an initial marking.

### 2.3. Cycle Time of TWMGs

The cycle time of a TWMG system $G=\langle N,\delta ,M_{0}\rangle$, denoted by χ(G), is the average period to fire one time the minimal T-semiflow $x=[x_{1},\ldots ,x_{m}]$ [28,34]. The steady evolution of a strongly connected TWMG system is repetitive. Let $f_{i}$ denote the firing frequency of transition $t_{i}$ during the repetitive period. Then, the cycle time of a TWMG system $G=\langle N,\delta ,M_{0}\rangle$ can be defined as:

(1) $$\chi \left(G\right)=\frac{x_{i}}{f_{i}},\forall t_{i}\in T.$$

We denote by $\chi_{\gamma }\left(G\right)$ the cycle time of elementary circuit γ and by $\chi_{\gamma^{*}}\left(G\right)=\max_{\gamma \in \Gamma }\chi_{\gamma }\left(G\right)$ the critical time. For a TMG system, the cycle time is equal to the critical time, i.e.,

$$\chi \left(G\right)=\chi_{\gamma^{*}}\left(G\right).$$

However, for a TWMG system, reference [35] shows that the cycle time is greater than or equal to the critical time, i.e.,

$$\chi \left(G\right)\geq \chi_{\gamma^{*}}\left(G\right).$$

The cycle time of a TWMG can be determined by the simulation. The work of [12,30] provides a lower bound and an upper bound of the cycle times that are easy to compute. However, in general, such approximations may be far from the actual cycle time and, hence, cannot be used as guidelines for performance optimization. On the other hand, a TWMG system G=〈N,δ,M〉 can be transformed into an equivalent place-timed marked graphs (PTMGs) system $\hat{G}=\langle \hat{N},\hat{\delta },\hat{M}\rangle$ as shown in Algorithm 1, which describes the same behavior, transition firing language, and cycle time [28]. Then, the cycle time χ(G) of the original TWMG system can be obtained by solving the following linear programming problem (LPP) for the equivalent PTMG system $\hat{G}$ [12,33]:

(2) $$\begin{array}{cc} \; & \min\;\chi (\hat{G})\\ s.t. & \hat{C}\cdot \alpha +\chi \left(\hat{G}\right)\cdot \hat{M}\geq D_{p}\cdot \hat{\mathrm{Post}}\cdot v \end{array}$$

where $\chi \left(G\right)=\chi \left(\hat{G}\right)\in R^{+}$, $\alpha \in R^{\hat{m}}$, $v={\vec{1}}_{\hat{m}\times 1}$ is the visit ratio vector, and $D_{p}\in N^{\hat{n}\times \hat{n}}$ is a diagonal matrix such that

(3) $$D_{p}\left(i,j\right)=\left\{\begin{array}{cc} \hat{\delta }\left(p_{i}\right), & \;\mathrm{if}\;i=j,\\ 0, & \;\mathrm{otherwise}. \end{array}\right.$$

Note that $t_{in(p_{j})}$, $t_{out(p_{j})}$, ⌊·⌋, and ⌈·⌉ in Algorithm 1 represent the unique input transition of place $p_{j}$, the unique output transition of place $p_{j}$, the floor operator, and the ceiling operator, respectively.
**Algorithm 1** Transformation of a TWMG into a PTMG [12].**Input:** A TWMG system G=〈N,δ,M〉 and the minimal T-semiflow $x={\left(x_{1},\ldots ,x_{m}\right)}^{T}$ of net *N*;**Output:** An equivalent PTMG system $\hat{G}=\langle \hat{N},\hat{\delta },\hat{M}\rangle$ such that $\chi \left(\hat{G}\right)=\chi \left(G\right)$;**for** each transition $t_{i}\in T$ **do**     delete $t_{i}$ and its corresponding arcs;    add $x_{i}$ transitions $t_{i}^{1}$, $t_{i}^{2}$, *…*, $t_{i}^{x_{i}}$ and $x_{i}$ places $q_{i}^{1}$, $q_{i}^{2}$, *…*, $q_{i}^{x_{i}}$ such that ${}^{•}q_{i}^{a}=t_{i}^{a}$, ${q_{i}^{a}}^{•}=t_{i}^{a\;\mathrm{mod}\;x_{i}+1}$, $\forall a=1,\ldots ,x_{i}$;    
(4)$$\mathrm{let}\left\{\begin{array}{l} \hat{M}\left(q_{i}^{1}\right)=\hat{M}\left(q_{i}^{2}\right)=\cdots =\hat{M}\left(q_{i}^{x_{i}-1}\right):=0,\\ \hat{M}\left(q_{i}^{x_{i}}\right):=1,\\ \hat{\delta }\left(q_{i}^{1}\right)=\hat{\delta }\left(q_{i}^{2}\right)=\cdots =\hat{\delta }\left(q_{i}^{x_{i}}\right):=0. \end{array}\right.$$**end for****for** each place $p_{j}\in P$ begin **do**    delete $p_{j}$ and its corresponding arcs;    let a:=0, s:=1;    **repeat**        
(5)$$b:=\left\lfloor \frac{M\left(p_{j}\right)+Post\left(p_{j},t_{in(p_{j})}\right)\cdot a}{Pre(p_{j},t_{out(p_{j})})}+1\right\rfloor$$
(6)$$\begin{array}{c} a:=\left\lceil \frac{Pre\left(p_{j},t_{out(p_{j})}\right)\cdot b-M\left(p_{j}\right)}{Post(p_{j},t_{in(p_{j})})}\right\rceil \end{array}$$        **if** $a\leq x_{in(p_{j})}$ **then**           Add place $p_{j}^{s}$ such that ${}^{•}p_{j}^{s}=t_{in(p_{j})}^{a}$, ${p_{j}^{s}}^{•}=t_{out(p_{j})}^{\left(b-1\right)\;\mathrm{mod}\;x_{out(p_{j})}+1}$           
(7)$$\hat{M}\left(p_{j}^{s}\right):=\left\lfloor \frac{b-1}{x_{out(p_{j})}}\right\rfloor$$
(8)$$\hat{\delta }\left(p_{j}^{s}\right):=\delta \left(t_{in(p_{j})}\right)$$           
(9)$$n_{j}:=s$$           s:=s+1        **end if**    **until** $a\geq x_{in(p_{j})}$**end for**

## 3. Problem Statement

In a TWMG that models a practical system, each transition $t_{i}$ models an operation performed by machines, robots, etc., which we call the servers. A server is modeled by a server place $p_{s_{i}}$ self-looped with $t_{i}$. We consider the performance optimization problem in TWMGs where both quantities and types of servers are variables to be simultaneously optimized. Precisely speaking:

- For each transition, $t_{i}$ with $k_{i}\in N_{>0}$ choices of server types, we use a $k_{i}$-component binary vector $Z_{i}={\left[Z_{i}\left(1\right),Z_{i}\left(2\right),\ldots ,Z_{i}\left(k_{i}\right)\right]}^{T}\in {\left\{0,1\right\}}^{k_{i}}$ to describe the server type selection of it, where
  $$Z_{i}\left(j\right)=\left\{\begin{array}{cc} 1, & \mathrm{the}\;j\mathrm{-th}\;\mathrm{type}\;\mathrm{of}\;\mathrm{server}\;\mathrm{is}\;\mathrm{selected},\\ 0, & \mathrm{otherwise}. \end{array}\right.$$
  Moreover, to ensure that the operation is consistent, we require that—among all of the $k_{i}$ types of servers of transition $t_{i}$—only one type can be selected, i.e.,    
  $$Z_{i}\left(1\right)+Z_{i}\left(2\right)+\cdots +Z_{i}\left(k_{i}\right)=1$$
- For each transition $t_{i}$, different servers have different performances (i.e., the firing delay) and costs. We use $\delta (t_{i},j)$ and $\lambda (t_{i},j)$ ($j\in \{1,\ldots ,k_{i}\}$) to denote the firing delay and the unit cost of server type *j*, respectively. We denote the firing delay vector of transition $t_{i}$ and the unit cost vector of transition $t_{i}$ by $\delta \left(t_{i}\right)={\left[\delta \left(t_{i},1\right),\delta \left(t_{i},2\right),\ldots ,\delta \left(t_{i},k_{i}\right)\right]}^{T}\in Q_{\geq 0}^{k_{i}}$, and $\lambda \left(t_{i}\right)={\left[\lambda \left(t_{i},1\right),\lambda \left(t_{i},2\right),\ldots ,\lambda \left(t_{i},k_{i}\right)\right]}^{T}\in Q_{\geq 0}^{k_{i}}$, respectively.
- For each transition $t_{i}$, we use $M(p_{s_{i}})$ to denote the server quantity of it, i.e., the number of servers to equip. Then, we use $M={\left[M\left(p_{s_{1}}\right),M\left(p_{s_{2}}\right),\ldots ,M\left(p_{s_{m}}\right)\right]}^{T}\in N^{m}$ to represent the server quantity of all servers in the gross TWMG system.

Therefore, the firing delay $\delta (t_{i})$ and the unit cost $\lambda (t_{i})$ of transition $t_{i}$ are determined by the following equation:

(10) $$\begin{array}{c} \lambda \left(t_{i}\right)=Z_{i}^{T}\cdot \lambda \left(t_{i}\right),\;\;\;\;\;\;\;\;\;\;\;\;\;\;\;\;\;\;\;\;\;\;\;\;\;\;\\ \delta \left(t_{i}\right)=Z_{i}^{T}\cdot \delta \left(t_{i}\right),\;\;\;\;\;\;\;\;\;\;\;\;\;\;\;\;\;\;\;\;\;\;\;\;\;\;\;\\ Z_{i}\left(1\right)+Z_{i}\left(2\right)+\ldots +Z_{i}\left(k_{i}\right)=1,\;\\ i=1,\ldots ,m.\;\;\;\;\;\;\;\;\;\;\;\;\;\;\;\;\;\;\;\;\;\;\;\;\;\;\;\;\;\;\;\;\;\;\;\;\; \end{array}$$

Moreover, the choices of server types and quantities must be subject to a given budget limit R∈R. Then, we can formulate the performance optimization problem as the following.

**Problem 1**  (Performance optimization problem). *Given a TWMG model N, a budget R∈R, a unit cost vector $\lambda (t_{i})$, and a firing delay vector $\delta (t_{i})$, determine a server-type selection $Z_{i}={\left[Z_{i}\left(1\right),Z_{i}\left(2\right),\ldots ,Z_{i}\left(k_{i}\right)\right]}^{T}$ and a server quantity $M={\left[M\left(p_{s_{1}}\right),M\left(p_{s_{2}}\right),\ldots ,M\left(p_{s_{m}}\right)\right]}^{T}$, such that $\lambda^{T}\cdot M\leq R$ while the cycle time χ(G) is minimized (i.e., the performance is maximized).*

In plain words, solving Problem 1 is equivalent to solving the following optimization problem

(11) $$\begin{array}{cc} \; & \min\;\chi (G)\\ s.t. & \left\{\begin{array}{c} \lambda^{T}\cdot M\leq R,\;\;\;\;\;\;\;\;\;\;\;\;\;\;\;\;\;\;\;\;\;\;\;\;\;\;\;\;\;\;\;\;\left(11a\right)\\ \left.\begin{array}{c} \lambda \left(t_{i}\right)=Z_{i}^{T}\cdot \lambda \left(t_{i}\right),\\ \delta \left(t_{i}\right)=Z_{i}^{T}\cdot \delta \left(t_{i}\right),\\ Z_{i}\left(1\right)+Z_{i}\left(2\right)+\ldots +Z_{i}\left(k_{i}\right)=1,\\ i=1,\ldots ,m, \end{array}\right\}\;\left(11b\right) \end{array}\right. \end{array}$$

where

- χ(G) represents the cycle time of the TWMG system G=〈N,δ,M〉,
- $\lambda ={\left[\lambda \left(t_{1}\right),\ldots ,\lambda \left(t_{m}\right)\right]}^{T}$ represents the cost vector of the TWMG,
- $M={\left[M\left(p_{s_{1}}\right),\ldots ,M\left(p_{s_{m}}\right)\right]}^{T}$ is the server quantity of the TWMG,
- $\delta ={\left[\delta \left(t_{1}\right),\ldots ,\delta \left(t_{m}\right)\right]}^{T}$ is the firing delay vector of the TWMG.

However, we note that solving this problem is challenging since it is a quadratic optimization: in general, a globally optimal solution is hard to achieve. Therefore, in the next section, we develop a hybrid method composed of genetic heuristics and MILP. The following example will be used as a running example in the sequel of this paper.

**Figure 1:**
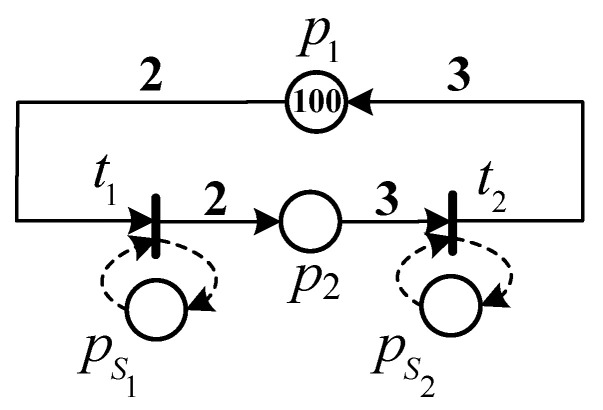
The TWMG model for Example 1.

**Example 1.**  *Consider the TWMG model N depicted in Figure 1 that represents a cyclic manufacturing system. It is composed of two different operations performed by machines ${\mathrm{MA}}_{1}$ and ${\mathrm{MA}}_{2}$, respectively. Transitions $t_{1}$ and $t_{2}$ represent the operation performed by machines ${\mathrm{MA}}_{1}$ and ${\mathrm{MA}}_{2}$, respectively. Place $p_{1}$ is an idle place that represents the raw material to proceed, place $p_{2}$ is an activity place that represents the manufacturing process, and places $p_{s_{1}}$ and $p_{s_{2}}$ are the server places corresponding to $t_{1}$ and $t_{2}$, respectively. Tokens in places $p_{s_{1}}$ and $p_{s_{2}}$ represent the numbers of machines ${\mathrm{MA}}_{1}$ and ${\mathrm{MA}}_{2}$, respectively. Initially, there are one hundred raw materials to be proceeded, i.e., $M(p_{1})=100$, and no semi-products are being produced, i.e., $M(p_{2})=0$.*

*Assume that we have $k_{1}=3,k_{2}=2$, i.e., transition $t_{1}$ has three choices of server types and transition $t_{2}$ has two choices. The firing delay and the unit cost of each type choice are listed in Table 1.*

$$\left\{\begin{array}{c} \lambda \left(t_{1}\right)={\left[\lambda \left(t_{1},1\right),\lambda \left(t_{1},2\right),\lambda \left(t_{1},3\right)\right]}^{T}={\left[4,10,15\right]}^{T},\\ \lambda \left(t_{2}\right)={\left[\lambda \left(t_{2},1\right),\lambda \left(t_{2},2\right)\right]}^{T}={\left[5,9\right]}^{T},\\ \delta \left(t_{1}\right)={\left[\delta \left(t_{1},1\right),\delta \left(t_{1},2\right),\delta \left(t_{1},3\right)\right]}^{T}={\left[15,4,2\right]}^{T},\\ \delta \left(t_{2}\right)={\left[\delta \left(t_{2},1\right),\delta \left(t_{2},2\right)\right]}^{T}={\left[20,18\right]}^{T}. \end{array}\right.$$

*The performance optimization problem (11) is to determine the quantities of servers of transitions $M={\left[M\left(p_{s_{1}}\right),M\left(p_{s_{2}}\right)\right]}^{T}$ and the server-type selections $Z_{1}=\left[Z_{1}\left(1\right),Z_{1}\left(2\right),Z_{1}\left(3\right)\right]$ and $Z_{2}=\left[Z_{2}\left(1\right),Z_{2}\left(2\right)\right]$ subject to a given budget R so that the cycle time of the system is minimized.*

## 4. Genetic MILP Approach for Performance Optimization in TWMGs

Due to the existence of weights on arcs of the TWMGs and the nonlinear constraint (11a), it is quite difficult to find an analytical approach to solve problem (11). Hence, in this section, we develop a hybrid method composed of genetic heuristics and mixed-integer linear programming, which makes the problem solvable.

As mentioned before, a possible server allocation strategy consists of two parts (M,Z) where M characterizes the choice of the quantity while $Z=(Z_{1},\ldots ,Z_{m})$ characterizes the choice of the server type. In our method, the two parts are optimized in an alternative manner. For small systems, it could be possible to enumerate the server type Z first and determine the quantity M subsequently. However, it is not possible for practical systems even with medium sizes. In addition, it was shown in [33] that for a TWMG whose server type Z is given, the optimal quantity M can be obtained by transforming it into a finite number of equivalent PTMG classes and solving a MILPP for each equivalent class. The computational cost of this approach is high since the number of equivalent PTMG classes increases exponentially w.r.t. the number of places of the original TWMG. In practice, it is inefficient to solve the performance optimization in TWMGs by exploring all the equivalent PTMGs.

**Figure 2:**
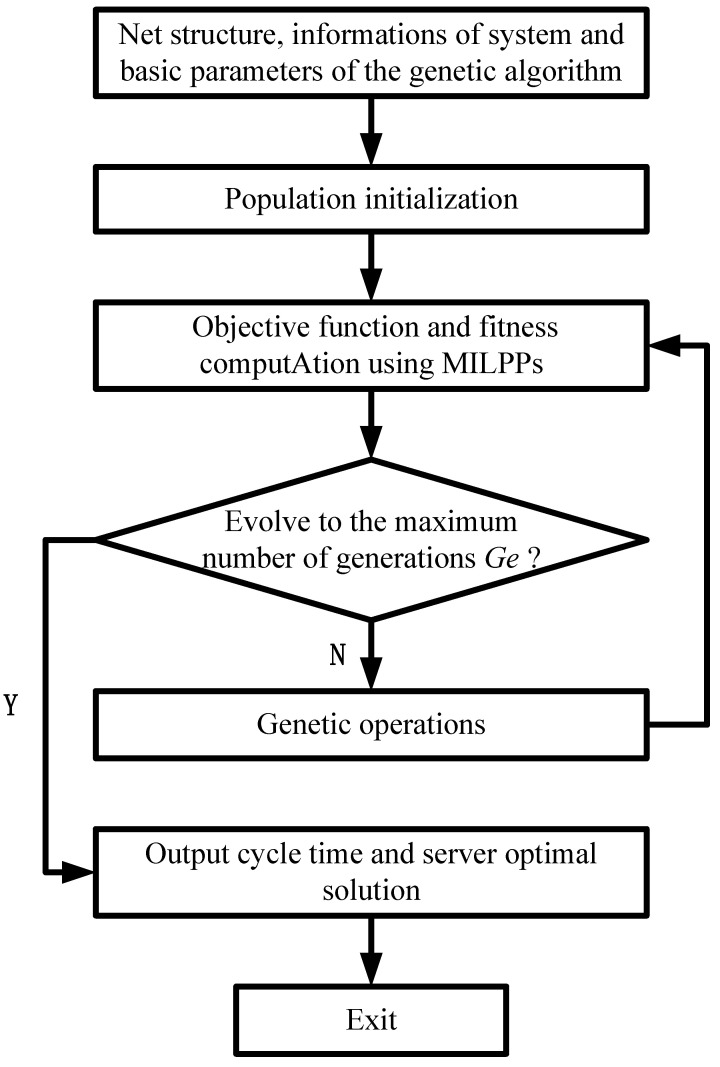
Flow chart of the developed approach based on genetic algorithm.

In this paper, the quantity-component M, i.e., the initial token distribution for server places in the TWMG, is first treated as a chromosome in the genetic heuristics. Then, among all the possible server types Z whose number grows exponentially with respect to the system size, we originally develop a MILPP to determine the optimal one. Therefore, the computational cost can be reduced significantly compared with the existing methods in [33]. Moreover, the result from the MILPP is also used to score the chromosome, i.e., the fitness of the quantity–component M, which provides a guideline for generating the next generation of candidate chromosomes. This alternated optimization is done when a pre-given number (Ge) that represents the maximum number of generation is reached. The main steps of the approach are sketched in the flow chart in Figure 2.

In the following subsections, we will discuss the main elements of the developed approach:

- Coding and decoding;
- Initial population generation;
- Calculation of the objective function and fitness value;
- The overall genetic algorithm: selection, crossover, and mutation.

### 4.1. Coding and Decoding

A server allocation strategy (i.e., server quantity) M can be coded as a chromosome Chm that is an *m*-dimensional nonnegative integer vector such that

(12) $$\mathrm{Chm}={\left[e_{1},e_{2},\ldots ,e_{m}\right]}^{T}=M,$$

where $e_{i}$ represents the quantities of servers of transition $t_{i}$, i.e., the number of tokens in the server place $p_{i}^{s}$. For instance, the chromosome of the TWMG model *N* depicted in Figure 1 can be expressed as $\mathrm{Chm}={\left[e_{1},e_{2}\right]}^{T}$, where $e_{1}=M\left(p_{1}^{s}\right)$ and $e_{2}=M\left(p_{1}^{2}\right)$. Therefore, we can decode Chm into a marking $M_{\mathrm{Chm}}={\left[4,0,e_{1},e_{2}\right]}^{T}$ of the TWMG model.

### 4.2. Population Generation and Feasibility Screening

In each iteration in the genetic algorithm, a set of candidate chromosomes are generated from promising ones in the previous generation. However, not all of the newly generated chromosomes—in our case the initial server distribution—are feasible. For example, some initial server distribution may result in the system eventually dying. Hence, a pre-screening step is performed to remove such unfeasible chromosomes from the pool of candidates.

First, a marking $M_{\mathrm{Chm}}$ that is decoded by a chromosome Chm should guarantee the liveness of a TWMG. A TWMG is live if and only if each elementary circuit is live [24]. Hence, a feasible chromosome necessarily satisfies the following condition:

(13) $$\left(\forall \gamma \in \Gamma \right)\;y^{T}\cdot M_{\mathrm{Chm}}>y^{T}\cdot M_{D}^{\gamma }$$

where y is the minimal T-semiflow corresponding to γ and $M_{D}^{\gamma }=[Pre\left(p_{1},p_{1}^{•}\right)-1,\ldots ,$$Pre\left(p_{n},p_{n}^{•}\right){-1]}^{T}$ is a marking restricted to γ.

On the other hand, a chromosome Chm is feasible only if there exists at least one server-type selection $Z_{i}=\left[Z_{i}\left(1\right),\ldots ,Z_{i}\left(k_{i}\right)\right]$ for every transition $t_{i}\in T$, such that the total cost of servers does not beyond the budget *R*. This indicates that the following constraint is necessarily feasible:

(14) $$\left\{\begin{array}{c} \lambda^{T}\cdot M\leq R,\\ M=\mathrm{Chm},\\ \lambda \left(t_{i}\right)=Z_{i}^{T}\cdot \lambda \left(t_{i}\right),\\ Z_{i}\left(1\right)+Z_{i}\left(2\right)+\ldots +Z_{i}\left(k_{i}\right)=1, \end{array}\right.$$

Combining the results in (13) and (14), both the liveness and feasibility of a chromosome Chm can be guaranteed by the following constraints:

(15) $$\left\{\begin{array}{c} y\cdot M_{\mathrm{Chm}}>y\cdot M_{D}^{\gamma },\forall \gamma \in \Gamma ,\\ \lambda^{T}\cdot M\leq R,\\ M=\mathrm{Chm},\\ \lambda \left(t_{i}\right)=Z_{i}^{T}\cdot \lambda \left(t_{i}\right),\\ Z_{i}\left(1\right)+Z_{i}\left(2\right)+\ldots +Z_{i}\left(k_{i}\right)=1, \end{array}\right.$$

where $M_{\mathrm{Chm}}$ is a marking decoded from chromosome Chm.

### 4.3. Objective Function and Fitness Score: A MILPP Approach

Given a chromosome Chm and its decoded marking $M_{\mathrm{Chm}}$ of a TWMG model *N*, we aim to determine the optimal server-type selection $Z=(Z_{1},\ldots ,Z_{m})$, such that the cycle time χ(G) of the TWMG system $G=\langle N,\delta ,M_{\mathrm{Chm}}\rangle$ is minimized while the cost of the servers corresponding to (M,Z) do not exceed the budget *R*.

As we discussed in Section 2.3, a TWMG system $G=\langle N,\delta ,M_{\mathrm{Chm}}\rangle$ with a known initial marking $M_{\mathrm{Chm}}$ can be transformed into an equivalent PTMG system $\hat{G}=\langle \hat{N},\hat{\delta },{\hat{M}}_{Chm}\rangle$ with the same cycle time. Hence, the cycle time χ(G) of *G* can be obtained by solving LPP (2) for its equivalent PTMG system $\hat{G}$. Note that here the firing delay vector $\delta ={\left[\delta \left(t_{1}\right),\ldots ,\delta \left(t_{m}\right)\right]}^{T}$ of *G* is dependent on the server-type selection Z which is undetermined yet. Hence, the firing delays in the corresponding equivalent PTMG system $\hat{G}$$\hat{\delta }=\left[\hat{\delta }\left(p_{1}\right),\ldots ,\hat{\delta }\left(p_{\hat{n}}\right)\right]$ is also to be determined. Now we show such an optimal server-type selection can be obtained by solving a MILPP for the equivalent PTMG.

**Proposition 1.**  *Given a performance optimization problem (11) for a TWMG system G whose equivalent PTMG system is $\hat{G}$, let $\left(\chi \left(\hat{G}\right),\;\alpha ,\;\lambda ,\;\delta ,\;\hat{\delta },\;D_{p},\;Z_{i}\right)$ be the optimal solution of the following MILPP:*

(16) $$\begin{array}{cc} \; & \min\;\chi (\hat{G})\\ s.t. & \left\{\begin{array}{l} \;\;\hat{C}\cdot \alpha +\chi \left(\hat{G}\right)\cdot {\hat{M}}_{\mathrm{Chm}}\geq D_{p}\cdot \hat{\mathrm{Post}}\cdot v,\;\;\;\;\;\;\;\;\;\;\;\;\;\left(16a\right)\\ \left.\begin{array}{c} \lambda^{T}\cdot M\leq R,\\ M=\mathrm{Chm}, \end{array}\right\}\;\;\;\;\;\;\;\;\;\;\;\;\;\;\;\;\;\;\;\;\;\;\;\;\;\;\;\;\;\;\;\;\;\;\;\;\;\;\;\;\;\;\;\;\;\;\;\;\left(16b\right)\\ \left.\begin{array}{c} \lambda \left(t_{i}\right)=Z_{i}^{T}\cdot \lambda \left(t_{i}\right),\\ \delta \left(t_{i}\right)=Z_{i}^{T}\cdot \delta \left(t_{i}\right), \end{array}\right\}\;\;\;\;\;\;\;\;\;\;\;\;\;\;\;\;\;\;\;\;\;\;\;\;\;\;\;\;\;\;\;\;\;\;\;\;\;\;\;\left(16c\right)\\ \;\;Z_{i}\left(1\right)+Z_{i}\left(2\right)+\ldots +Z_{i}\left(k_{i}\right)=1,\;\;\;\;\;\;\;\;\;\;\;\;\;\;\;\;\;\;\;\;\;\;\left(16d\right)\\ \;\;i=1,\ldots ,m,\;\;\;\;\;\;\;\;\;\;\;\;\;\;\;\;\;\;\;\;\;\;\;\;\;\;\;\;\;\;\;\;\;\;\;\;\;\;\;\;\;\;\;\;\;\;\;\;\;(16e)\\ \left.\begin{array}{c} \hat{\delta }\left(q_{i}^{a}\right)=0,\;a=1,\ldots ,x_{i},\\ \hat{\delta }\left(p_{j}^{s}\right)=\delta \left(t_{q}\right),\;\forall^{•}p_{j}=t_{q},\;s=1,\ldots ,n_{j} \end{array}\right\}\;\;\;\;\;\;\;\;\;\;\;\;\left(16f\right) \end{array}\right. \end{array}$$

*Then, $Z_{i}$ (i=1,…,m) is an optimal server-type selection of problem (11) with respect to a given server quantity M=Chm.*

**Proof.**  Constraint (16b) ensures that the choice of server types subject to server quantity M=Chm does not exceed the budget *R*. Constraints (16c), (16d), and (16e) jointly enforce Equation (10). Combining the results in Equations (2) and (6), constraint (16f) ensures the correctness of the firing delay equivalence between the original TWMG and the PTMG. According to the results in [12,33], constraint (16a) can provide an optimal solution for performance optimization if $\hat{C}$ and $M_{\mathrm{Chm}}$ are given. Therefore, $Z_{i}$ (i=1,…,m) is an optimal server-type selection of problem (11) restricted to a given server quantity M=Chm.    □

Given a chromosome Chm, the cycle time $\chi (\hat{G})$ in the optimal solution of MILPP (16) is denoted as $F\left(M_{Chm}\right)=\chi^{*}$. Let $N_{i}$ denote the pool of chromosomes in the *i*-th iteration, i.e., the *i*-th generation, we define

$$F_{min}=\min_{\mathrm{Chm}\in N_{i}}F\left(\mathrm{Chm}\right)$$

and

$$F_{max}=\max_{\mathrm{Chm}\in N_{i}}F\left(\mathrm{Chm}\right)$$

the minimum and maximum objective functions (i.e., cycle time) of the population in the current generation, respectively. Then, the fitness of a chromosome Chm is defined as

(17) $$fitness\left(\mathrm{Chm}\right)=1-\frac{F\left(\mathrm{Chm}\right)-F_{min}}{F_{max}-F_{min}}$$

**Figure 3:**
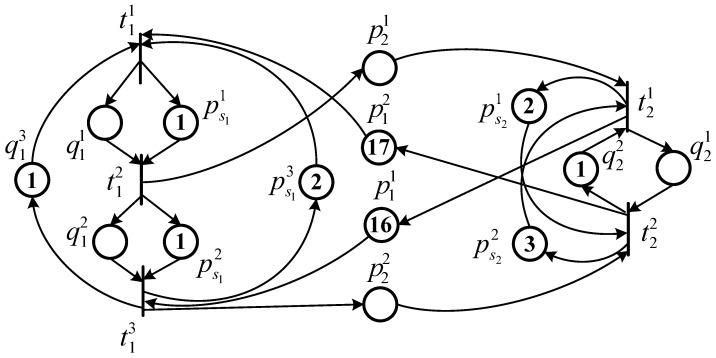
The equivalent PTMG corresponding to the TWMG model *N* in Figure 1 with an initial marking $M_{\mathrm{Chm}}=\left[100,0,4,5\right]$.

**Example 2.**  *Let us consider again the TWMG model N discussed in Example 1 with a minimal T-semiflow $x={\left[3,2\right]}^{T}$ and a chromosome $\mathrm{Chm}={\left[4,5\right]}^{T}$. Then, the decoded initial marking is $M_{Chm}={\left[100,0,4,5\right]}^{T}$. The equivalent PTMG system $\hat{G}=\langle \hat{N},\hat{\delta },{\hat{M}}_{Chm}\rangle$ corresponding to $M_{Chm}$ is shown in Figure 3, where the firing delay vector $\hat{\delta }$ is to be determined.*

*Let R=100 be the budget limit. According to Proposition 1, a MILPP can be established as shown in Equation (18). By solving MILPP (18), we obtain $Z=(Z_{1},Z_{2})$ where $Z_{1}={\left[0,1,0\right]}^{T}$ and $Z_{2}={\left[0,1\right]}^{T}$. Thus, to optimize the performance of the PTMG (which is equal to that of the original TWMG), transition $t_{1}$ should be equipped with the second type of server while transition $t_{2}$ should be equipped with the second type. The optimal cycle time $\chi \left(G\right)=\chi \left(\hat{G}\right)=7.2$ of the TWMG system $G=\langle N,,\delta ,M_{\mathrm{Chm}}\rangle$. Finally, the objective function of chromosome $\mathrm{Chm}={\left[4,5\right]}^{T}$ is F(Chm)=7.2, and its fitness can be computed according to Equation (17).*

(18) $$\begin{array}{c} \min\;\chi (\hat{G})\\ s.t.\left\{\begin{array}{c} \hat{C}\cdot \alpha +\chi \left(\hat{G}\right)\cdot {\hat{M}}_{\mathrm{Chm}}\geq D_{p}\cdot \hat{\mathrm{Post}}\cdot v,\\ \left.\begin{array}{c} \lambda^{T}\cdot M\leq R,\\ M=\mathrm{Chm}, \end{array}\right\}\\ \left.\begin{array}{c} \lambda \left(t_{1}\right)=Z_{1}\left(1\right)\cdot \lambda \left(t_{1},1\right)+Z_{1}\left(2\right)\cdot \lambda \left(t_{1},2\right)+Z_{1}\left(3\right)\cdot \lambda \left(t_{1},3\right),\\ \delta \left(t_{1}\right)=Z_{1}\left(1\right)\cdot \delta \left(t_{1},1\right)+Z_{1}\left(2\right)\cdot \delta \left(t_{1},2\right)+Z_{1}\left(3\right)\cdot \delta \left(t_{1},3\right),\\ \lambda \left(t_{2}\right)=Z_{2}\left(1\right)\cdot \lambda \left(t_{2},1\right)+Z_{2}\left(2\right)\cdot \lambda \left(t_{2},2\right),\\ \delta \left(t_{2}\right)=Z_{2}\left(1\right)\cdot \delta \left(t_{2},1\right)+Z_{2}\left(2\right)\cdot \delta \left(t_{2},2\right), \end{array}\right\}\\ \left.\begin{array}{c} Z_{1}\left(1\right)+Z_{1}\left(2\right)+Z_{1}\left(3\right)=1,\\ Z_{2}\left(1\right)+Z_{2}\left(2\right)=1, \end{array}\right\}\\ \;\;i=1,2,\\ \left.\begin{array}{c} \hat{\delta }\left(q_{1}^{1}\right)=0,\hat{\delta }\left(q_{1}^{2}\right)=0,\hat{\delta }\left(q_{1}^{3}\right)=0,\\ \hat{\delta }\left(q_{2}^{1}\right)=0,\hat{\delta }\left(q_{2}^{2}\right)=0,\\ \hat{\delta }\left(p_{1}^{1}\right)=\delta \left(t_{2}\right),\hat{\delta }\left(p_{1}^{2}\right)=\delta \left(t_{2}\right),\\ \hat{\delta }\left(p_{2}^{1}\right)=\delta \left(t_{1}\right),\hat{\delta }\left(p_{2}^{2}\right)=\delta \left(t_{1}\right),\\ \hat{\delta }\left(p_{s_{1}}^{1}\right)=\delta \left(t_{1}\right),\hat{\delta }\left(p_{s_{1}}^{2}\right)=\delta \left(t_{1}\right),\\ \hat{\delta }\left(p_{s_{1}}^{3}\right)=\delta \left(t_{1}\right),\\ \hat{\delta }\left(p_{s_{2}}^{1}\right)=\delta \left(t_{2}\right),\hat{\delta }\left(p_{s_{2}}^{2}\right)=\delta \left(t_{2}\right), \end{array}\right\} \end{array}\right. \end{array}$$

### 4.4. The Overall Genetic Algorithm

Now we are ready to introduce the overall genetic algorithm in our approach.

#### 4.4.1. Selection

The purpose of the selection operation is to save chromosomes with higher fitness values in the parent population and to eliminate chromosomes with low fitness values. The selection rule we used here combines the classical roulette wheel selection [43] (also called the proportional selection, which ensures the diversity of genes inherited by the next generation) and the optimal retention [43] (that can effectively retain the optimal individual of each generation). In a roulette selection, the probability of each chromosome Chm to be selected is proportional to its fitness value fitness(Chm). In generation *i* with population $N_{i}$, the probability of selecting a chromosome Chm is given by:

(19) $$Prob\left({\mathrm{Chm}}_{i}\right)=\frac{fitness(\mathrm{Chm})}{\sum_{{\mathrm{Chm}}_{j}\in N_{i}}fitness\left({\mathrm{Chm}}_{j}\right)}.$$

Note that it may eliminate chromosomes with large fitness values during the roulette wheel selection operation. Therefore, we retain the chromosome with the highest fitness value through the optimal retention method before the roulette wheel selection operation.

#### 4.4.2. Crossover

In the genetic algorithm, new chromosomes are generated through the crossover operation by exchanging gene segments from two parent chromosomes to find better offspring. In the literature, several crossover operations have been proposed, such as single-point crossover, multi-point crossover, and uniform crossover [43]. In this paper, we used a single-point crossover operation for the genetic algorithm.

#### 4.4.3. Mutation

The mutation operation is to randomly—usually with a pre-set probability—perturb one or several genes of a chromosome. The mutation operation is of great significance in the genetic algorithm to maintain population diversity, prevent falling into the local optimal solution, and enrich the gene pool. The mutation operation uses site mutation, i.e., one or more genes of a chromosome are randomly selected and changed with a mutation probability. In this paper, we use the single-point mutation operation, i.e., at most one gene in each chromosome is mutated for each operation.

Note that during the crossover operation and mutation operation, a newly generated chromosome Chm may not always satisfy the constraints in Equation (15), i.e., the TWMG may not be live at the decoded marking $M_{\mathrm{Chm}}$, or the total cost of servers corresponding to $M_{\mathrm{Chm}}$ exceeds the budget. In such a case, the chromosome that leads to an infeasible solution is immediately discarded, and a fully randomized chromosome, which satisfies the constraint (15) is added to the pool.

The entire hybrid method that combines genetic heuristics with MILP is presented as Algorithm 2, whose inputs are: a TWMG model *N*, the budget *R*, the unit cost vector $\lambda (t_{i})$, the firing delay vector $\delta (t_{i})$, the population size $N_{p}$ (i.e., N), the maximum number of generations Ge, the crossover probability $P_{c}$, and the mutation probability $P_{m}$.
**Algorithm 2** Genetic MILP method for performance optimization in TWMGs.**Input:** A TWMG model *N*, R∈R, $\lambda \left(t_{i}\right)\in Q_{\geq 0}^{m}$, $\delta \left(t_{i}\right)\in Q_{\geq 0}^{m}$, $N_{p}\in N$, Ge∈N, $P_{c}\in R_{\geq 0}$, $P_{m}\in R_{\geq 0}$, $T_{0}\in R_{\geq 0}$, $T_{e}\in R_{\geq 0}$, and $\varepsilon \in R_{\geq 0}$;**Output:** Server-type selection $Z={\left[Z_{1},\ldots ,Z_{m}\right]}^{T}$ and server quantity $M={\left[M\left(p_{s_{1}}\right),\ldots ,M\left(p_{s_{m}}\right)\right]}^{T}$;**while**k≤Ge **do**    **for** $j=1,\ldots ,N_{p}$ **do**        Transform $\langle N,M_{{\mathrm{Chm}}_{j}^{k}}\rangle$ into $\langle \hat{N},{\hat{M}}_{{\mathrm{Chm}}_{j}^{k}}\rangle$ according to Algorithm 1;        Solve MILPP (16) for $\langle \hat{N},{\hat{M}}_{{\mathrm{Chm}}_{j}^{k}}\rangle$;        Calculate $fitness({\mathrm{Chm}}_{j}^{k})$ according to Equation (17);    **end for**    Let $j^{*}=arg\max_{{\mathrm{Chm}}_{j}^{k}\in N_{k}}fitness\left({\mathrm{Chm}}_{j}^{k}\right)$;    Let $M:=M_{Chm_{j^{*}}^{k}}$ be the server quantity and $Z:=(Z_{1},\ldots ,Z_{m})$ be the server type selection corresponding to chromosome ${\mathrm{Chm}}_{j^{*}}^{k}$;    **if** k+1≤Ge **then**        k:=k+1;        Execute selection, crossover, and mutation to generate an offspring generation $N_{k}=\{{\mathrm{Chm}}_{1}^{k},$$\ldots ,{\mathrm{Chm}}_{N_{p}}^{k}\}$;    **else**        Output M and Z    **end if****end while**

## 5. Illustrative Examples

In this section, the developed approach in Section 4 is illustrated by investigating the TWMG model discussed in Example 1 and a real flexible manufacturing system. The algorithms are implemented by MATLAB 2017 with YALMIP R20181012 subroutines on a computer installed Windows 10 operating system with CPU Intel Core i5 at 3.0 GHz and RAM 8 GB.

### 5.1. First Example

**Example 3.**  *Let us continue Example 1 and assume that the upper bound on the cost of servers is R=100. Parameters in the developed approach are set as the population size $N_{p}=10$, the crossover probability $P_{c}=0.7$, and the mutation probability $P_{m}=0.1$.*

*In Table 2, we show the best solution (i.e., the minimal cycle time), the worst solution (i.e., the maximal cycle time), the average solution, and the average CPU time for the developed approach (i.e., genetic MILP) by testing a different maximum number of generations Ge. For each generation, we executed the developed approach ten times. An optimal/suboptimal solution is found as follows:*

$$\begin{array}{c} M={\left[2,14\right]}^{T},\;Z_{1}={\left[0,0,1\right]}^{T},\;Z_{2}={\left[1,0\right]}^{T}, \end{array}$$

*which means that the optimal quantity of server of $t_{1}$ and $t_{2}$ are 2 and 14, respectively, while the third type of server of $t_{1}$ and the first type of server of $t_{2}$ are selected. By enumerating all possible server-type selections and server quantities, we found that the solutions obtained by the proposed approach are optimal. In addition, we also implemented the developed approach without MILP (i.e., genetic in Table 2), which means that both quantities and types of servers are generated by the genetic algorithm. It can be observed that the qualities of the solutions are improved by using the MILP we developed.*

### 5.2. Application to a Real Flexible Manufacturing System

In this subsection, we apply the developed approach to a hydraulic torque converter production line that is taken from [36]. The hydraulic torque converter is assembled with four different products as shown in Table 3. The production process of these products include 25 operations operated on 17 different machines (denoted by ${\mathrm{MA}}_{1},\ldots ,{\mathrm{MA}}_{17}$). We mention that the turbine is also assembled by two different products. Note that some machines are shared by one or more production lines, such as ${\mathrm{MA}}_{1}$, ${\mathrm{MA}}_{3}$, ${\mathrm{MA}}_{4}$, and ${\mathrm{MA}}_{6}$.

**Figure 4:**
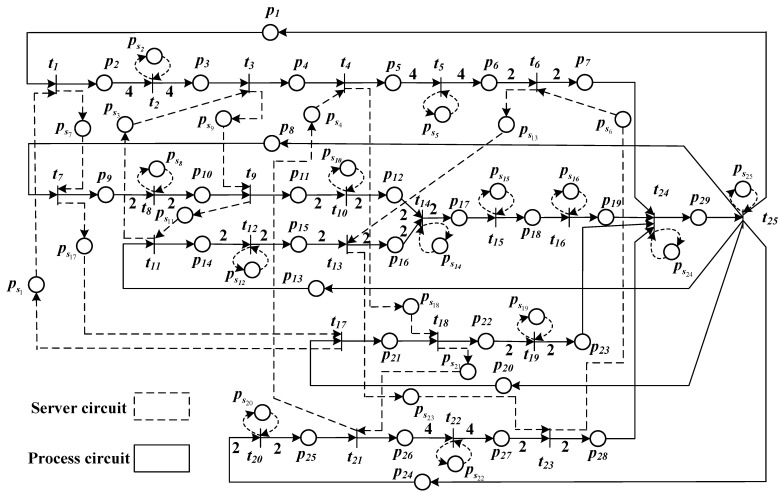
The TWMG model of the flexible manufacturing system.

The TWMG model is shown in Figure 4, which has 25 transitions and 54 places. It is composed of three different types of elementary circuits, i.e., process circuits that represent the manufacturing process, server circuits that represent the usage of machines, and mixed circuits that consist of both process circuits and server circuits. Tokens in places $p_{1}$, $p_{8}$, $p_{13}$, $p_{20}$, and $p_{24}$ represent the raw materials for each production line that are assumed to be 1000, i.e., $M(p_{j})=1000$ (j=1,8,13,20,24).

In Table 4, we present the unit costs of servers and the firing delays of the corresponding transitions. The budget is set to R=300. Simulation results for different cases are presented in Table 5. Parameters in the developed approach are the same as in Example 3.

For all of the cases, we execute each approach ten times. Since the best solution for the maximum number of generations Ge=5 is identical to the solution obtained for generation Ge=10, there is no need to test more generations for the genetic MILP approach. An optimal/suboptimal solution is found by the developed approach with the cycle time χ(G)=68, and the server-type selection and server quantity are as follows:

$$\begin{array}{c} M={\left[1,1,1,2,1,1,2,1,2,1,1,2,1,2,1,1,1,1,1,1,1,1,0,1,1\right]}^{T},\\ Z_{1}\left(1\right)=1,Z_{2}\left(1\right)=1,Z_{3}\left(3\right)=1,Z_{4}\left(1\right)=1,Z_{5}\left(1\right)=1,\\ Z_{6}\left(5\right)=1,Z_{7}\left(1\right)=1,Z_{8}\left(1\right)=1,Z_{9}\left(3\right)=1,Z_{10}\left(1\right)=1,\\ Z_{11}\left(3\right)=1,Z_{12}\left(1\right)=1,Z_{13}\left(5\right)=1,Z_{14}\left(1\right)=1,Z_{15}\left(1\right)=1,\\ Z_{16}\left(2\right)=1,Z_{17}\left(1\right)=1,Z_{18}\left(1\right)=1,Z_{19}\left(1\right)=1,Z_{20}\left(2\right)=1,\\ Z_{21}\left(1\right)=1,Z_{22}\left(1\right)=1,Z_{23}\left(5\right)=1,Z_{24}\left(3\right)=1,Z_{25}\left(1\right)=1. \end{array}$$

Note that due to the shared usage of resources, some different operations (transitions) are executed by the same machine ${\mathrm{MA}}_{i}$ (i=1,3,4,6). Therefore, the server quantities of machine ${\mathrm{MA}}_{i}$ equals the sum of the server quantities of all the transitions corresponding to ${\mathrm{MA}}_{i}$; the server types of all the transitions corresponding to ${\mathrm{MA}}_{i}$ should be identical.

$$\begin{array}{c} \mathrm{Server}\;\mathrm{quantity}\;\mathrm{of}\;{\mathrm{MA}}_{1}:M\left(p_{s_{1}}\right)+M\left(p_{s_{7}}\right)+M\left(p_{s_{17}}\right)=4,\\ \mathrm{Server}\;\mathrm{quantity}\;\mathrm{of}\;{\mathrm{MA}}_{3}:M\left(p_{s_{3}}\right)+M\left(p_{s_{9}}\right)+M\left(p_{s_{11}}\right)=4,\\ \mathrm{Server}\;\mathrm{quantity}\;\mathrm{of}\;{\mathrm{MA}}_{4}:M\left(p_{s_{4}}\right)+M\left(p_{s_{18}}\right)+M\left(p_{s_{21}}\right)=4,\\ \mathrm{Server}\;\mathrm{quantity}\;\mathrm{of}\;{\mathrm{MA}}_{6}:M\left(p_{s_{6}}\right)+M\left(p_{s_{13}}\right)+M\left(p_{s_{23}}\right)=2. \end{array}$$

From the simulation results, we can conclude that in the case of the same maximum number of generations, the cycle time (i.e., the objective function) obtained by the genetic MILP approach is smaller than that of the Genetic approach, i.e., the solution obtained by the genetic approach is improved by using the MILP.

We conclude this section by discussing the previous works. The approaches developed in [33,34,35,36] can provide optimal or near-optimal solutions under the condition that the types of resources are given. In this paper, we further extend these works by assuming that the types of resources are variables that also have to be optimized. Therefore, the existing approaches in [33,34,35,36] cannot provide a solution for the problem considered in this paper.

## 6. Conclusions

In this paper, we study the performance optimization for a class of PNs to maximize the throughput of the system subject to a given budget, while both quantities and typesof servers are decision variables. A genetic algorithm combined with mixed-integer linear programming was originally developed to obtain a near-optimal solution. The developed approach is based on linear programming techniques and provides an efficient solution for solving the performance optimization problem where both quantities and types of resources are simultaneously optimized. Our future work will focus on exploring an analytical solution for the considered optimization problem. On the other hand, we will consider improving the efficiency of the developed approach by using some advanced hybrid methods [44].

## Figures and Tables

**Table 1:** The unit cost and the firing delays of each server on the transitions for Example 1.

Transition	Server Type	Unit Cost of Server Type *j*	Firing Delay
$t_{i}$	*j*	$\lambda (t_{i},j)$	$\delta (t_{i},j)$ [s]
$t_{1}$	1	4	15
	2	10	4
	3	15	2
$t_{2}$	1	5	20
	2	9	18

**Table 2:** Simulation results of Example 3.

	Approaches	Genetic MILP				Genetic			
Obtained Results									
Maximal number of generation Ge		1	2	5	15	1	2	5	15
Best solution		3.0	3.0	3.0	3.0	3.3	3.3	3.3	3.1
Worst solution		4.5	4.0	3.0	3.0	6.0	5.0	5.0	4.0
Average solution		3.7	3.2	3.0	3.0	4.4	4.2	3.7	3.7
Average CPU time [s]		7.5	11.0	20.5	59.4	3.1	4.6	9.1	24.2

**Table 3:** Production process of the flexible manufacturing system.

Pump wheel	${\mathrm{MA}}_{1}$→${\mathrm{MA}}_{2}$→${\mathrm{MA}}_{3}$→${\mathrm{MA}}_{4}$→${\mathrm{MA}}_{5}$→${\mathrm{MA}}_{6}$
Turbine	${\mathrm{MA}}_{1}$→${\mathrm{MA}}_{7}$→${\mathrm{MA}}_{3}$→${\mathrm{MA}}_{8}$→${\mathrm{MA}}_{10}$→${\mathrm{MA}}_{11}$→${\mathrm{MA}}_{12}$
	${\mathrm{MA}}_{3}$→${\mathrm{MA}}_{9}$→${\mathrm{MA}}_{6}$→${\mathrm{MA}}_{10}$→${\mathrm{MA}}_{11}$→${\mathrm{MA}}_{12}$
Guide wheel	${\mathrm{MA}}_{1}$→${\mathrm{MA}}_{4}$→${\mathrm{MA}}_{13}$
Cover wheel	${\mathrm{MA}}_{14}$→${\mathrm{MA}}_{4}$→${\mathrm{MA}}_{15}$→${\mathrm{MA}}_{6}$
Hydraulic torque converter	${\mathrm{MA}}_{16}$→${\mathrm{MA}}_{17}$

**Table 4:** Unit costs of the servers and the firing delays of each operation.

Transition	Machine	Server Type	Unit Cost of Server	Firing Delay	Transition	Machine	Server Type	Unit Cost of Server	Firing Delay
$t_{i}$	MA	*j*	$\lambda (t_{i},j)$	$\delta (t_{i},j)$ [s]	$t_{i}$	MA	*j*	$\lambda (t_{i},j)$	$\delta (t_{i},j)$ [s]
$t_{1}$	${\mathrm{MA}}_{1}$	1	7	27	$t_{13}$	${\mathrm{MA}}_{6}$	2	7	34
		2	15	19			3	8	29
		3	20	15			4	10	26
		4	23	13			5	11	20
$t_{2}$	${\mathrm{MA}}_{2}$	1	4	25	$t_{14}$	${\mathrm{MA}}_{10}$	1	10	45
		2	8	22			2	14	38
$t_{3}$	${\mathrm{MA}}_{3}$	1	7	40	$t_{15}$	${\mathrm{MA}}_{11}$	1	8	17
		2	8	37			2	12	13
		3	9	25	$t_{16}$	${\mathrm{MA}}_{12}$	1	5	18
$t_{4}$	${\mathrm{MA}}_{4}$	1	8	16			2	9	12
		2	9	15	$t_{17}$	${\mathrm{MA}}_{1}$	1	9	15
		3	10	9			2	15	14
		4	12	6			3	20	10
$t_{5}$	${\mathrm{MA}}_{5}$	1	3	29			4	23	6
		2	6	25	$t_{18}$	${\mathrm{MA}}_{4}$	1	8	18
		3	8	20			2	9	16
$t_{6}$	${\mathrm{MA}}_{6}$	1	6	38			3	10	16
		2	7	35			4	12	13
		3	8	32	$t_{19}$	${\mathrm{MA}}_{13}$	1	13	24
		4	10	28			2	17	19
		5	11	25			3	20	13
$t_{7}$	${\mathrm{MA}}_{1}$	1	9	26	$t_{20}$	${\mathrm{MA}}_{14}$	1	7	38
		2	15	23			2	11	31
		3	20	18	$t_{21}$	${\mathrm{MA}}_{4}$	1	8	25
		4	23	12			2	9	22
$t_{8}$	${\mathrm{MA}}_{7}$	1	2	31			3	10	20
		2	5	26			4	12	14
		3	7	20	$t_{22}$	${\mathrm{MA}}_{15}$	1	10	36
$t_{9}$	${\mathrm{MA}}_{3}$	1	7	33			2	14	30
		2	8	25	$t_{23}$	${\mathrm{MA}}_{6}$	1	6	37
		3	9	16			2	7	32
$t_{10}$	${\mathrm{MA}}_{8}$	1	2	11			3	8	29
		2	7	8			4	10	24
$t_{11}$	${\mathrm{MA}}_{3}$	1	7	26			5	11	20
		2	8	22	$t_{24}$	${\mathrm{MA}}_{16}$	1	14	58
		3	9	10			2	21	42
$t_{12}$	${\mathrm{MA}}_{9}$	1	5	47			3	30	20
		2	8	42	$t_{25}$	${\mathrm{MA}}_{17}$	1	7	13
		3	13	35			2	8	10
$t_{13}$	${\mathrm{MA}}_{6}$	1	6	35			3	12	6

**Table 5:** Simulation results of the developed approach for the flexible manufacturing system.

	Approaches	Genetic MILP				Genetic					
Obtained Results											
Maximal number of generation Ge		1	2	5	10	2	5	10	40	70	100
Best solution		76.0	72	68.0	68.0	130.0	102.0	102.0	90.0	90.0	90.0
Worst solution		84.0	77.3	76.0	76.0	212.0	204.0	180.0	168.0	130.0	130.0
Average solution		79.7	75.2	71.3	70.6	171.8	159.4	152.2	126.2	120.8	111.7
Average CPU time [s]		72.9	152.9	396.5	1059.3	4.7	12.7	28.8	142.8	433.3	843.8

## Data Availability

Not applicable.

## Abbreviations

The following abbreviations are used in this manuscript:

PN	Petri net
FMS	flexible manufacturing system
MILP	mixed-integer linear programming
TMG	timed marked graph
TWMG	timed weighted marked graph
MILPP	mixed-integer linear programming problem
WMG	weighted marked graph
MG	marked graph
TPN	timed Petri net
TTPN	transition-timed Petri net
PTPN	place-timed Petri net
PTMG	place-timed marked graph
